# Structural, Electrical and Corrosion Properties of Bulk Ti–Cu Alloys Produced by Mechanical Alloying and Powder Metallurgy

**DOI:** 10.3390/ma17071473

**Published:** 2024-03-23

**Authors:** Katarzyna Arkusz, Kamila Pasik, Marek Nowak, Mieczyslaw Jurczyk

**Affiliations:** 1 Department of Biomedical Engineering, Faculty of Mechanical Engineering, University of Zielona Gora, 9 Licealna Street, 65-417 Zielona Gora, Poland; k.arkusz@iimb.uz.zgora.pl (K.A.); k.pasik@iimb.uz.zgora.pl (K.P.); 2 Institute of Materials Science and Engineering, Faculty of Materials Engineering and Technical Physics, Poznan University of Technology, 24 Jana Pawla II Street, 61-138 Poznan, Poland; marek.nowak@put.poznan.pl

**Keywords:** titanium, Ti–Cu alloys, mechanical alloying, electrochemical properties, corrosion resistance, biosensors

## Abstract

Binary Ti_100-x_–Cu_x_ (x = 1.6 and 3.0 wt.%) alloys were produced by the application of mechanical alloying and powder metallurgy processes. The influence of the copper concentration in titanium on the microstructure and properties of bulk alloys was investigated. The synthesized materials were characterized by an X-ray diffraction technique, scanning electron microscopy, and chemical composition determination. The electrochemical and corrosion properties were also investigated. Cold compaction and sintering reduced the content of α-Ti content in Ti98.4–Cu1.6 and Ti97–Cu3 alloys to 92.4% and 83.7%, respectively. Open Circuit Potential measurements showed a positive shift after the addition of copper, suggesting a potential deterioration in the corrosion resistance of the Ti–Cu alloys compared to pure Ti. Electrochemical Impedance Spectroscopy analysis revealed significant improvement in electrical conductivity after the addition of copper. Corrosion testing results demonstrated compromised corrosion resistance of Ti–Cu alloys compared to pure Ti. In summary, the comprehensive investigation of Ti_100-x_–Cu_x_ alloys provides valuable insights for potential applications in biosensing.

## 1. Introduction

Titanium and its alloys are commonly used for hard tissue implants. Their properties include high biocompatibility, corrosion resistance in body fluids, osseointegration, and high relative strength [1,2,3,4]. Advancements in electronics and microfabrication techniques have generated growing interest in the application of implantable biosensors in precision medicine.

TiO_2_ stands out among transition metal oxides due to its superior conductivity and high biocompatibility. It is recognized as a promising material, being an N-type semiconductor oxide with a 3d24s2 electronic configuration in the outermost shell. The four valence electrons in TiO_2_ form covalent bonds with oxygen atoms, ensuring high chemical stability. With a band gap of approximately 3.2 eV, the sensing characteristics of TiO_2_ can be enhanced by introducing additional energy levels to the conduction band through doping or imperfections [5,6,7].

TiO_2_ has recently found applications in various biosensing tasks, including the detection of glucose, cholesterol, troponin, and cancer diagnosis [8,9,10,11]. However, the electrical and electrochemical properties of TiO_2_ have to be improved by doping more conductive elements/structures like gold nanoparticles, copper nanoparticles, graphene, etc., to improve detection performance. Due to the non-stable connection between Ti-based electrodes and conductive additives, developing new, homogeneous materials with high electrical conductivity is recommended. Due to the high conductivity of copper (5.96 · 10^7^ S/m at 20 °C) Ti–Cu-type materials seem to be promising in biosensing.

Many attempts have been made to create Ti–Cu-type biomaterials [12,13,14,15,16,17]. According to the phase diagram of Ti–Cu, the maximum solubilities of Cu in (α-Ti) and (β-Ti) are 1.6 and 13.5 at.% at 790 and 1005 °C, respectively. At room temperature, the solid solubility of copper in α-Ti is negligibly small [13]. Extended solid solubility by mechanical alloying approaches was reported earlier in several alloy systems [18,19].

Ti–Cu alloys have broad application prospects in the biomedical field due to their excellent properties. The properties of Ti–Cu alloys were strongly dependent on Cu content, microstructures, their Ti_2_Cu phase, and their preparation process. The effect of Cu content on the precipitation behaviors and the mechanical and corrosion properties of the as-cast Ti–Cu alloys was studied recently by Wang et al. [17]. The volume fraction of Ti_2_Cu phases affected the electrochemical performances of the alloys.

Ti–Cu alloys with different Cu contents (3, 5, and 7 wt.%) were fabricated by arc-melting [12]. These alloys with the microstructure of α-Ti + Ti_2_Cu showed the best ductility compared with other Ti–Cu alloys. These results indicated that the Ti–Cu alloys with the microstructure of α-Ti + Ti_2_Cu showed the best ductility compared with other Ti–Cu alloys with microstructures of α-Ti + transformed β-Ti and completely transformed β-Ti. The increase in the Cu content significantly contributed to the decreased ductility due to the increasing amount of Ti_2_Cu, which brought both solid solution strengthening and precipitation strengthening [12]. Additionally, the Ti–5Cu alloy showed excellent antibacterial properties and corrosion resistance.

One of the other methods of Ti-based alloy powder synthesis was mechanical alloying (MA), which allows obtaining biomaterials [18,19]. MA can be controlled by some parameters like milling time, ball-to-powder mass ratio (BPR), milling atmosphere and temperature, mill type, milling speed, etc. [18,19]. Improved material properties due to the nanocrystalline or ultrafine structure transition were observed. Hardness improvement can be detected in synthesized biomaterials due to the grain boundary strengthening mechanism [20,21]. In published studies, metal surfaces utilizing low-micron to nanophase topography demonstrated increased adhesion of osteoblasts [22,23]. 

Amorphous Ti_1-x_–Cu_x_ (0.10 < x ≤ 0.87) [24] and Ti_x_–Cu_100-x_ (x = 90, 80, 70, and 60) alloys were synthesized by using a high-energy ball mill [25], and their microstructure and amorphous phases were studied. For example, the Ti80Cu20 alloys were obtained in an amorphous state after 30 h of MA, and the amorphous phase was stable up to 340 °C. At higher temperatures, the crystallization of the amorphous phase produced an intermetallic compound, Ti_2_Cu, and α-Ti [25]. Crystalline Ti–Cu alloys showed good mechanical properties and biocompatibility [26,27], as well as good bio-corrosion [28]. Additionally, the as-cast Ti–Cu alloys showed a higher hardness and mechanical strength as well as a higher antibacterial rate but lower corrosion resistance in comparison to titanium metal. On the other hand, annealing at 900 °C/2 h increased the hardness and strength and improved the corrosion resistance but had a small influence on the antibacterial property [28]. It was shown that the Ti_2_Cu phase played a key role in the antibacterial mechanism.

The relationship between the temperature of spark plasma sintering and the characteristics of the Ti–Cu material has been established recently [16]. The mechanical properties increase due to the phase composition changes as a function of an increase in the temperature of the sintering process.

In this study, crystalline Ti–Cu alloys of 1.6 and 3.0 wt.% Cu were produced using the MA method. The resulting material was in powder form and was formed into bulk samples by cold pressing and sintering. Electrochemical properties of the Ti–Cu alloys with different Cu content were studied through Open Circuit Potential (OCP) measurements, electrochemical impedance spectroscopy, and polarization resistance measurements. Each characteristic was performed in 0.01 M phosphate-buffered saline and Ringer solution.

## 2. Materials and Methods

This paper describes the research results of a study carried out on Ti_100-x_–Cu_x_ (x = 1.6 and 3.0 wt.%) alloys synthesized by MA in an argon atmosphere using the powder metallurgy method.

### 2.1. Materials and Reagents

Powders of titanium (<45 μm, 99.9%,) and copper (53–88 μm, 99.9%) were purchased from Alfa Aesar (Haverhill, MA, USA). Phosphate-buffered saline (0.01 M PBS, 0.0027 M potassium chloride, and 0.137 M sodium chloride pH 7.4) was purchased from Sigma-Aldrich (St. Louis, MO, USA). Ringer solution was prepared by dissolving one tablet (Merck, no 115525) in 500 mL neutral deionized water and then sterilized in an autoclave (15 min at 121 °C). The final solution (500 mL) contained NaCl—1.125 g, KCl—0.0525 g, anhydrous CaCl_2_—0.03 g, and NaHCO_3_—0.025 g, with a pH value in the range of 6.8–7.2 at 25 °C. All solutions of chemical substances were prepared with Milli-Q water.

### 2.2. Sample Preparation

Powders of α-Ti and Cu were used for the synthesis of alloys. The Ti and Cu powders were weighed, blended, and inserted into stainless steel vials in a glove box (LabMaster 130, National Institute of Standards and Technology, Gaithersburg, MD, USA) filled with automatically controlled argon atmosphere (O_2_ < 2 ppm and H_2_O < 1 ppm). The MA was performed under Ar (99.999% purity) by the application of the SPEX 8000 Mixer Mill (SPEX SamplePrep, Metuchen, NJ, USA). MA lasted 7 h. The ratio of hard steel ball weight (12 mm diameter) to powder weight equaled 6:1. As-milled materials were finally cold-pressed at a pressure of 1.4 GPa and heat-treated at 1000 °C for 1 h under high-purity argon atmosphere with 2% hydrogen; heating and cooling of the samples took place together with the furnace.

### 2.3. Materials Characterization

The crystallographic structure evolution of the samples during the synthesis process was studied at room temperature using X-ray diffraction (XRD) with a Panalytical Empyrean diffractometer with CuKα (λ = 1.54056 Å) radiation (Almelo, The Netherlands). Rietveld analysis [29] was applied to calculate the lattice constants and phase quantity using Malvern Panalytical B.V. HighScore 5.2 version with Plus option software. α-Ti (ref. code 01-071-4632), Ti_2_Cu (ref. code 04-003-2231), and TiO (ref. code 01-086-2352) were used as structural models.

A scanning electron microscope (SEM, Tescan MIRA3, Brno, Czech Republic) with an energy dispersive spectrometer (EDS, ULTIM MAX Oxford Instruments, Abingdon, UK) was applied to characterize the chemical composition and element distribution of the elements in the alloys. The density of the bulk sintered alloys was calculated by the Archimedes method. For the sample porosity measurement, the formula P = (1 − *ρ*/*ρ*_th_) × 100% was used, where *ρ* is the density of the porous material, and *ρ*_th_ is its corresponding theoretical density calculated based on the rule of mixtures.

The electrochemical experiments were performed in the three-electrode system using an Autolab PGSTAT302N (Metrohm, Herisau, Switzerland) in 0.01 M PBS and Ringer solution. The working electrode was commercial pure Ti and Ti_100-x_–Cu_x_ alloy, the counter electrode was a platinum mesh, and the reference electrode was a silver chloride electrode (E_Ag/AgCl_ = 0.222 V vs. standard hydrogen electrode). The OCP was measured for 1800 s. Electrochemical Impedance Spectroscopy (EIS) was recorded over the frequency range of 0.1 Hz to 10^5^ Hz (10 frequency steps/decade) with an excitation voltage of 10 mV. The EIS results were analyzed by fitting the experimental impedance data with electrical equivalent circuit models using NOVA 2.1 software. The criteria for assessing the fitting quality included considering the lower chi-squared value and the lower estimative errors (in %) for all components.

Potentiodynamic polarization curves were obtained by changing the electrode potential in the range of −1 to 1 V against Ag/AgCl with a scan rate of 1 mV/s. The corrosion potentials (E_corr_) and anodic and cathodic Tafel slopes (b_a_ and b_c_) were calculated from the polarization curves using the linear extrapolation method. The linear polarization resistance (R_p_) was determined by the slope of the current–potential plot in the range of 2 mV about the corrosion potential. Then, the corrosion current density (I_corr_) and the corrosion rate (ν_corr_) were calculated using the Stern–Geary equation.

Electrochemical studies (OCP, EIS, Tafel) were conducted with a minimum of 5-fold repeatability.

## 3. Results and Discussion

### 3.1. Crystallography and Microstructure

**Figure 1:**
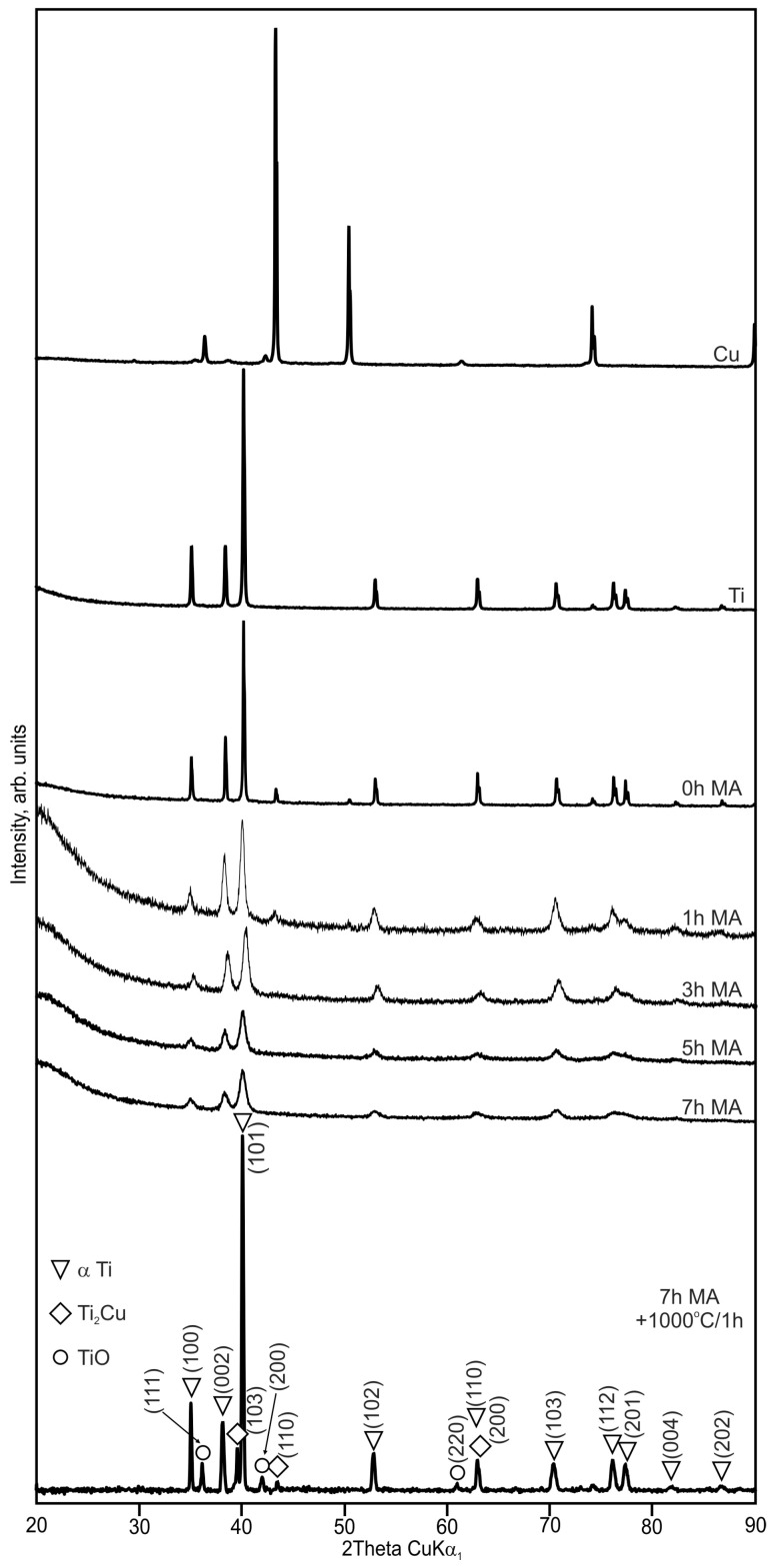
X-ray diffraction (XRD) spectra of Ti98.4–Cu1.6 powders mechanically alloyed (MA) for different times (0, 1, 3, 5, and 7 h) and bulk alloy sintered at 1000 °C/1 h.

**Figure 2:**
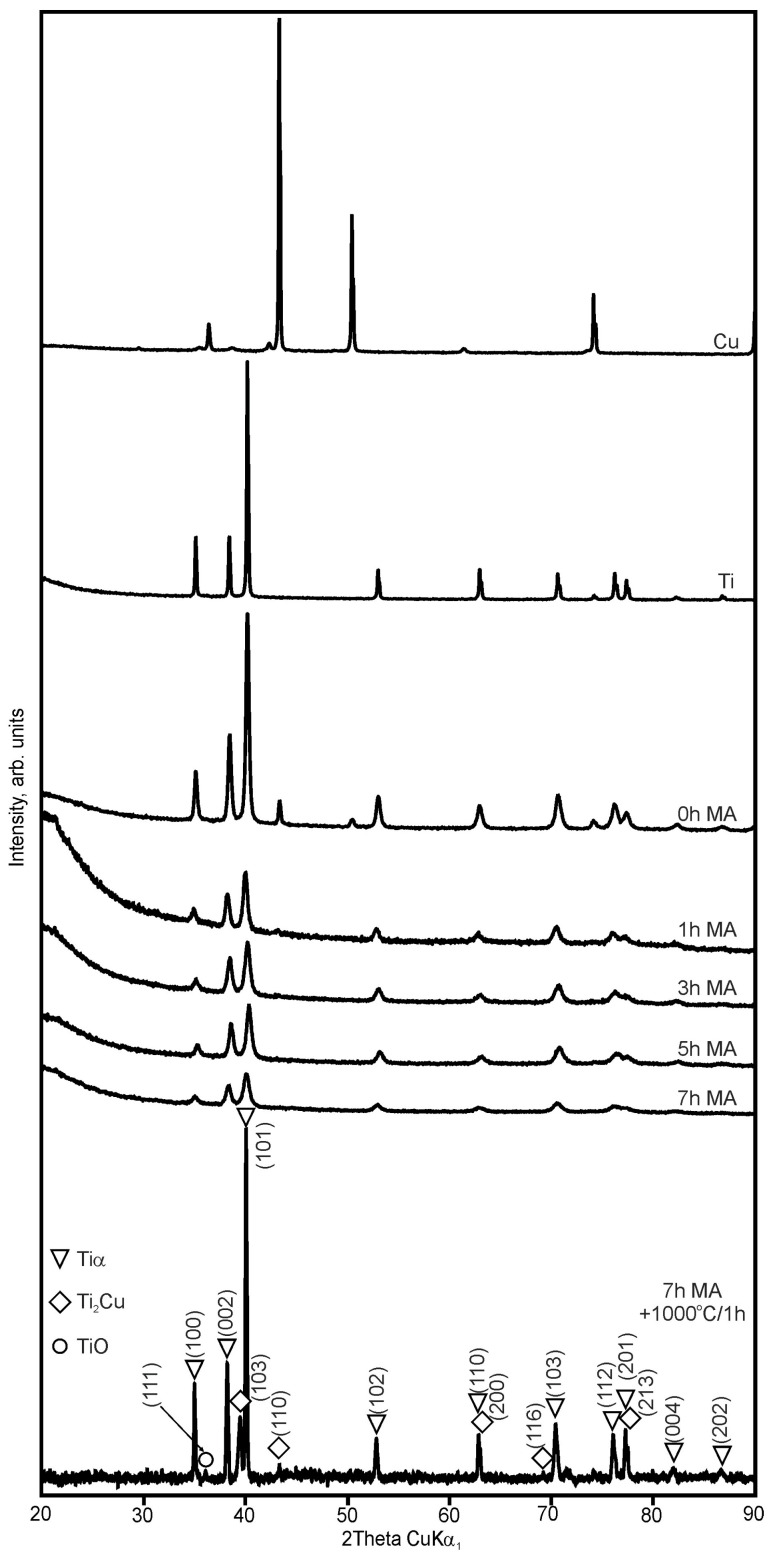
XRD spectra of Ti97–Cu3 powders mechanically alloyed for different times (0, 1, 3, 5, and 7 h) and bulk alloy sintered at 1000 °C/1 h.

The synthesis of the Ti_100-x_–Cu_x_ (x = 1.6 and 3.0 wt.%) alloys by MA and powder metallurgy method was the aim of the current study. The crystal structure changes during MA of the Ti_100-x_–Cu_x_ system were studied in detail (Figure 1 and Figure 2). The typical (hkl) indexes of the copper were not visible after 1 h of MA. After 7 h of milling, only the α-Ti phase was visible. During processing, an energy transfer to a powdered material results in an increase in the density of defects with a subsequent subgrain formation. In some cases, depending on the composition of the starting chemical composition of the alloy, an amorphization can occur [19].

**Figure 3:**
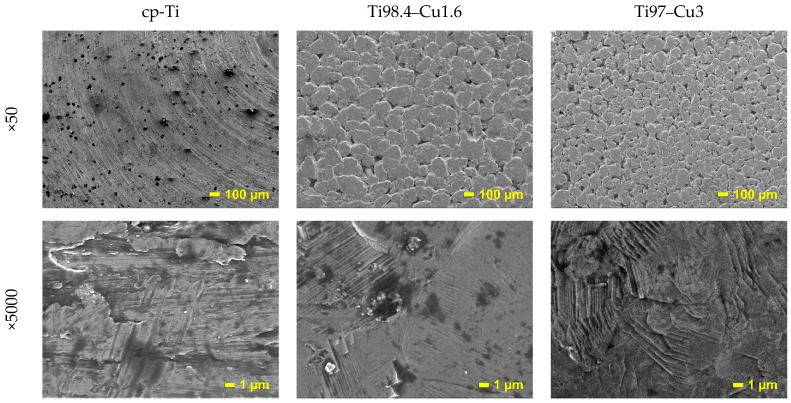
SEM micrographs of commercial pure α-Ti (cp-Ti) and bulk Ti_100-x_–Cu_x_ (x = 1.6, 3.0 wt.%) alloys.

The cold compaction and sintering of Ti_100-x_–Cu_x_ MA powders did not cause the formation of the single-phase α-Ti-type structures (Figure 1 and Figure 2). The amount of α-Ti decreased to 92.4 and 83.7% in the Ti98.4–Cu1.6 and Ti97–Cu3 alloys, respectively. Except the Ti_2_Cu phase, Ti oxide (TiO) was detected by the X-ray method for both alloys (Table 1 and Table 2). The crystallite sizes estimated by the Williamson–Hall UDM (Uniform Deformation Model) approach of MA for 7 h and sintered at 1000 °C for 1 h were close to 165 nm for Ti98.4–Cu1.6 and 270 nm for Ti97–Cu3, respectively. The theoretical density (*ρ*_th_), the calculated density of the synthesized alloys (*ρ*_cal_), and porosity (P) are shown in Table 3. The porosity of the obtained alloys by cold-pressing and sintered at 1000 °C/1 h approach was 13%; see Figure 3.

**Figure 4:**
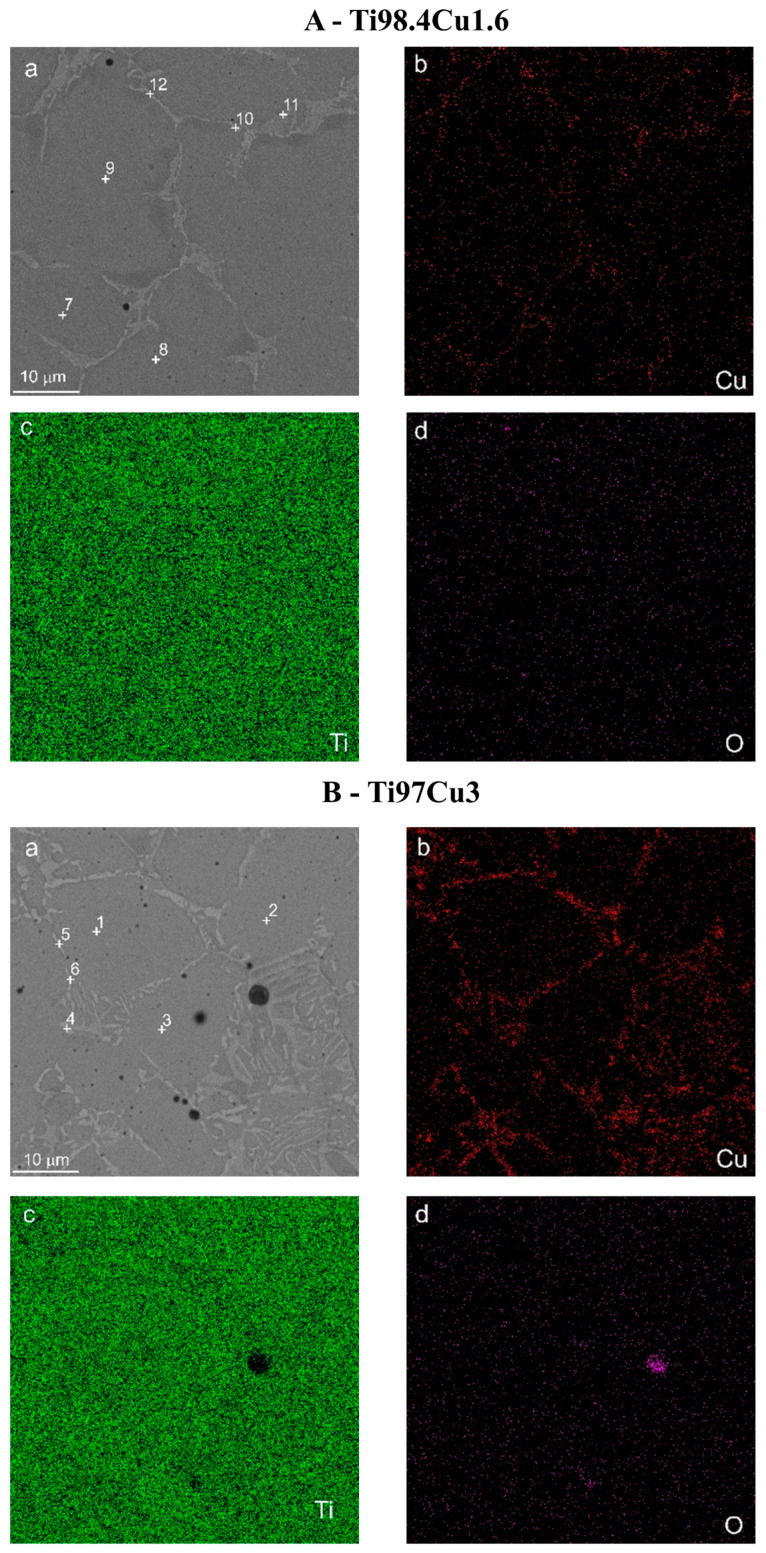
SEM photo of Ti98.4–Cu1.6 (**A**) and Ti97–Cu3 (**B**) alloys with energy dispersive spectrometer (EDS) analysis spots (1–12) marked (**a**)—see Table 4; (**b**–**d**) EDS maps of element distributions in the alloy.

The SEM image of the commercial pure α-Ti (cp-Ti) surface sample was equivalent to the mechanically polished surface represented by the typical morphology of native oxide film, with a thin and non-porous structure (Figure 3). This native oxide film was spontaneously formed on the Ti surface on exposure to air at room temperature [30]. The results of the EDS analysis of the distribution of elements on the surface of polished samples of Ti_100-x_–Cu_x_ alloys (x = 1.6, 3.0 wt.%) are shown as pictures in Figure 4 and as the chemical composition in Table 4. The obtained results indicate that the structure of both alloys consists of the basic α-Ti phase and the second Ti_2_Cu phase located at the grain boundaries. The analysis of Rietveld method phase participation showed that the Ti_2_Cu phase was 7.1 and 16% for x = 1.6 and 3.0 wt.%, respectively. EDS analysis also showed the presence of oxygen in Ti_100-x_–Cu_x_ samples (Table 4). This was confirmed by X-ray analysis which showed the presence of Ti oxides (TiO) (Figure 1 and Figure 2).

### 3.2. Electrochemical Properties

**Figure 5:**
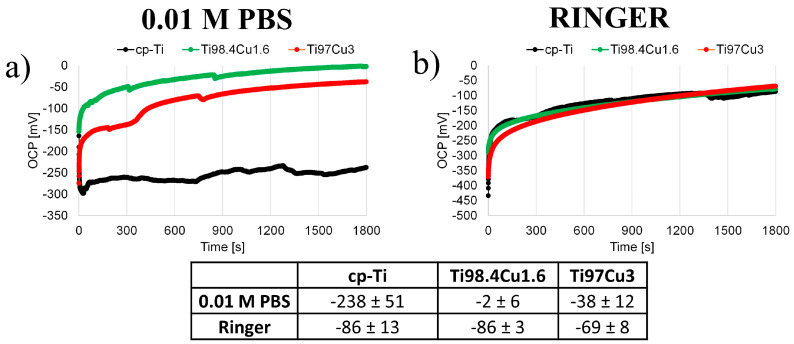
Open Circuit Potential of cp-Ti and Ti_100-x_–Cu_x_ (x = 1.6, 3.0 wt.%) alloys measured in 0.01 M PBS (**a**) and Ringer solution (**b**) for 1800 s.

The corrosion potential, the potential of the samples in relation to the reference electrode, was recorded in an open circuit for a duration of 1800 s in 0.01 M PBS (Figure 5a) and Ringer solution (Figure 5b). The final potential recorded during this period was considered as the corrosion potential and is listed in Figure 5. The corrosion potentials of Ti_100-x_–Cu_x_ (x = 0, 1.6, 3.0 wt.%) alloys measured in Ringer solution (Figure 5b) closely approximate each other, measuring approximately −86–−69 mV. In contrast, the corrosion potential of commercially pure Ti measured in 0.01 M PBS is lower, with a value of −238 mV, compared to Ti98.4–Cu1.6 and Ti97–Cu3, with values of −2 mV and −38 mV, respectively. The presence of copper in the examined surfaces results in shifts in the E_corr_ for more positive values.

**Figure 6:**
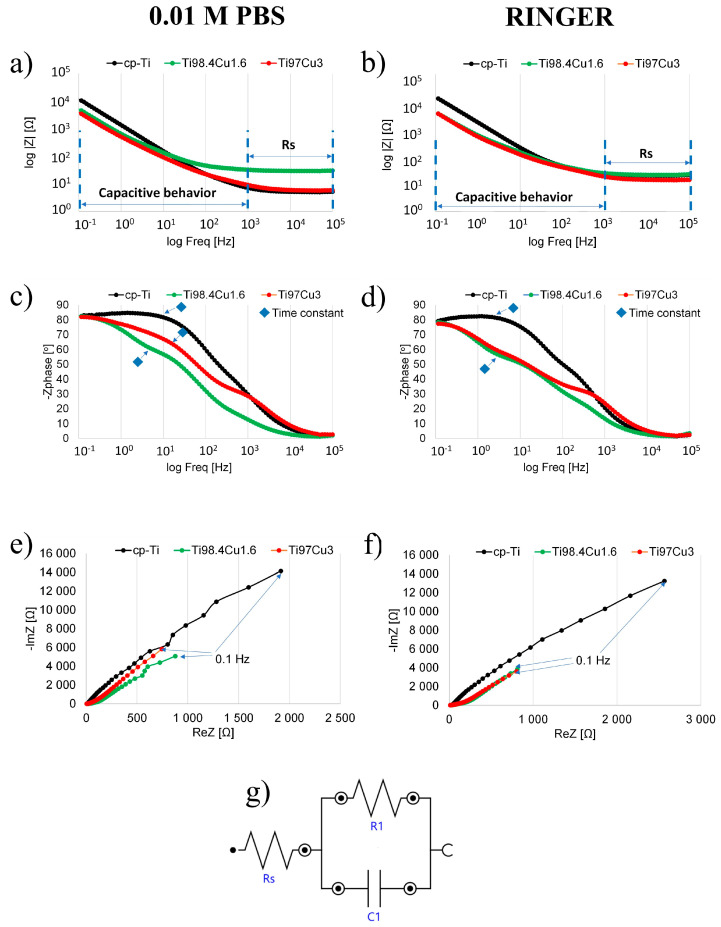
Bode (**a**–**d**) and Nyquist (**e**,**f**) plots of cp-Ti and Ti_100-x_–Cu_x_ (x = 1.6, 3.0 wt.%) alloys measured in 0.01 M PBS (**a**,**c**,**e**) and Ringer solution (**b**,**d**,**f**), and equivalent circuit (**g**).

Based on the SEM images (Figure 3 and Figure 4), the passive film developed on the surfaces of α-Ti and Ti–Cu alloys is anticipated to possess a double-layer structure consisting of an inner barrier layer and an outer hydroxide layer. Examination of the Bode magnitude plot (Figure 6a,b) reveals that the modulus of impedance |Z| remained constant in the high-frequency range, spanning from 10^5^ Hz down to 10^3^ Hz, with the phase angle approaching 0° (as depicted in the Bode phase plot, Figure 6c,d). This behavior indicates a resistive nature corresponding to the solution resistance between the working and reference electrodes [31]. In the PBS and Ringer medium and low-frequency range (from 10^3^ to 10^−1^), the Bode phase plot (Figure 6b) exhibited one-time constants within the frequency range of 10^0^ to 10^2^ Hz [32,33,34]. This observation aligns with the anticipated one-layer structure of the passive film.

Ti98.4–Cu1.6 and Ti97–Cu3 showed a linear relationship in the Bode magnitude plot (Figure 6a,b) within the same medium (0.01 M PBS and Ringer) and low-frequency range (from 100 to 10^−1^). Simultaneously, the phase angle values (Figure 6c,d) approached 83° and 82° in PBS and 78° and 77° in Ringer, respectively. These EIS results correspond to the expected capacitive behavior for titanium and Ti–Cu alloys in the nearly capacitive region [33,35]. This behavior is attributed to the response of the highly stable passive film. As explained by Li et al. [36], changes in the capacitive behavior can be discerned by analyzing the values of phase angle approaching 90° in the low-frequency region i.e., 0.1 Hz, indicating the presence of a highly compact oxide film.

The corrosion resistance of the Ti97–Cu3 alloy can be directly inferred from the radius of the capacitive-semi arcs in the Nyquist representation (Figure 6e,f). In Figure 6e, the depressed size of the arc following the addition of 1.6 and 3.0 wt.% Cu indicates a decrease in charge transfer resistance. Obtained EIS results were fitted to the Electric Equivalent Circuit (EEC) shown in Figure 6g. Within this EEC, Rs represents the resistance of the phosphate-buffered saline solution, R1 denotes the resistance of the barrier layer [22], and C1 corresponds to the impedance of the double-layer passive film [31,37]. These electric elements represent the double layer, including charge transfer resistances and capacitances of the inner-barrier and outer-hydroxide layers.

The values for the circuit parameters for the Ti_100-x_–Cu_x_ alloys (x = 0, 1.6, and 3.0 wt.%) are presented in Table 5, derived from fitting the EEC to the experimental EIS data using NOVA software. The magnitudes of resistances for the barrier layer decrease with increasing copper concentration. Reducing the resistance of the electrical double layer resulted in the sample surface exhibiting lower resistance to the infiltration of corrosive ions, such as Cl^−^, onto the TiCu substrate [34]. The magnitudes of capacitances for the barrier layer increase with the concentration of copper. These observations from the EIS fitting are consistent in 0.01 M PBS and Ringer solution and align with findings in existing literature [31].

**Figure 7:**
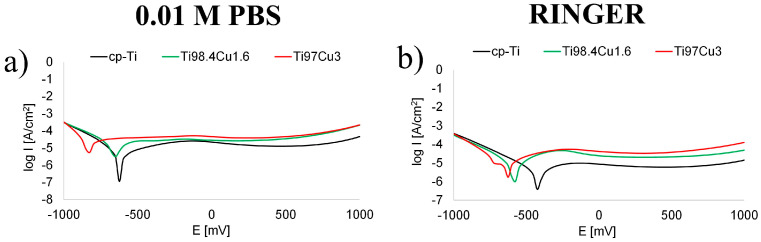
Potentiodynamic polarization curves for cp-Ti and Ti_100-x_–Cu_x_ (x = 1.6, 3.0 wt.%) alloys measured in (**a**) 0.01 M PBS and (**b**) Ringer solution.

Table 6 shows the calculated values of the corrosion potential (E_corr_), corrosion current density (I_corr_), and corrosion rate (ν_corr_) obtained from the polarization plot represented in Figure 7. Corrosion parameters were calculated based on the potential values of the cathodic and anodic regions from the Tafel plot. Increasing the Cu content in Ti_100-x_–Cu_x_ resulted in decreasing E_corr_ and increasing I_corr_ measured in 0.01 M PBS and Ringer solution. The extrapolated values of corrosion rate indicate a shift toward higher current densities. The negative shift in E_corr_ and the increase in I_corr_ affirm that the corrosion rate magnifies with the incorporation of copper into the Ti alloys.

Obtained values of the corrosion parameter were similar to other results in the literature indicating that E_corr_ of α-Ti measured in 0.01 M PBS was –0.60 V [38] and E_corr_ of α-Ti measured in Ringer solution was −421 [39]. The decrease in corrosion performance by the addition of copper was also confirmed in another study [38]. However, it should be noted that the corrosion rates were still within the acceptable range (0.02–0.13 mm/y) for biocompatibility of metal [40].

## 4. Conclusions

The bulk Ti_100-x_–Cu_x_ alloys (x = 1.6 and 3.0 wt.%) were produced by the application of MA and powder metallurgy. Various research methods, including detailed studies of crystal structure changes during MA, microstructure analyses, and electrochemical and corrosion investigations, were applied.

Microstructure analysis revealed the dynamic nature of the MA process, evident in the disappearance of characteristic (hkl) copper indexes after 1 h of milling, and the dominance of the α-Ti phase after 7 h. Despite several attempts, cold compaction and sintering did not lead to the formation of a single-phase α-Ti structure, reducing the α-Ti content to 92.4% and 84% in Ti98.4–Cu1.6 and Ti97–Cu3 alloys, respectively.

OCP measurements showed a positive shift after the addition of copper, suggesting a potential deterioration in the corrosion resistance of the alloys compared to pure Ti. EIS analysis revealed significant changes in impedance modules, especially after the addition of copper. A substantial reduction in the impedance module was observed, indicating increased susceptibility to the penetration of corrosive ions and higher electric conductivity in biosensing. Corrosion testing results demonstrated compromised corrosion resistance of Ti–Cu alloys compared to pure Ti, particularly for the Ti97–Cu3 alloy.

In summary, the comprehensive investigation of Ti_100-x_–Cu_x_ alloys, including synthesis methods, crystal structure changes, and corrosion behavior, provides valuable insights for potential applications in biosensing. Future studies can further explore the optimization of alloy compositions and surface modification for enhanced performance in biological environments.

## Figures and Tables

**Table 1:** Crystal structure and lattice parameters of phases present in the synthesized alloys.

Phases	Structure	Lattice Parameters
α-Ti	Hexagonal P63/mmc	a (Å): 2.953 c (Å): 4.709
Ti_2_Cu	Tetragonal I4/mmm	a (Å): 2.939 c (Å): 10.728
TiO	Cubic Fm-3m	a (Å): 4.325

**Table 2:** Results of phase abundance analysis in Ti_100-x_–Cu_x_ (x = 1.6, 3.0 wt.%) alloys by the Rietveld method.

	Phase Fractions %		
Composition	α-Ti	Ti_2_Cu	TiO
Ti98.4–Cu1.6	92.4	7.1	0.5
Ti97–Cu3	83.7	16.0	0.3

**Table 3:** Theoretical density (*ρ*_th_), the calculated density of the porous materials (*ρ*_cal_) and porosity (P) of bulk Ti_100-x_–Cu_x_ (x = 1.6, 3.0 wt.%) alloys.

Composition	*ρ*_th_ [g/cm^3^]	*ρ*_cal_ [g/cm^3^]	P [%]
Ti98.4–Cu1.6	4.5545	3.9280	13.8
Ti97–Cu3	4.5966	3.9874	13.3

**Table 4:** Results of spot EDS analysis of the chemical composition of the studied alloys.

Composition	EDS Analysis Point Number	Ti		Cu		O	
		wt.%	Σ	wt.%	σ	wt.%	σ
Ti97–Cu3	Results obtained for the inner areas of the grains						
	1	92.2	0.4	1.2	1.2	6.6	0.4
	2	92.9	0.4	1.4	0.2	5.7	0.4
	3	92.7	0.4	1.1	0.2	6.2	0.4
	Results obtained for grain boundaries						
	4	64.6	0.3	31.5	0.3	3.9	0.3
	5	66.9	0.3	25.3	0.3	4.8	0.3
	6	66.6	0.3	28.4	0.3	5.0	0.3
Ti98.4–Cu1.6	Results obtained for the inner areas of the grains						
	7	93.1	0.4	0.7	0.2	6.2	0.4
	8	92.8	0.4	0.7	0.2	6.5	0.4
	9	92.9	0.2	1.2	0.2	5.9	0.4
	Results obtained for grain boundaries						
	10	71.7	0.3	24.0	0.3	4.2	0.3
	11	70.4	0.3	24.8	0.3	4.7	0.3
	12	72.4	0.3	22.6	0.3	5.0	0.3

**Table 5:** EIS fitting results for cp-Ti and Ti_100-x_–Cu_x_ (x = 1.6 and 3.0 wt.%) alloys measured in 0.01 M PBS and Ringer solution using the equivalent circuit shown in Figure 6g.

EC Parameter	cp-Ti		Ti98.4–Cu1.6		Ti97–Cu3	
	Value	SD	Value	SD	Value	SD
0.01 M PBS						
Rs [Ohm∙cm^2^]	46.15	1.99	72.77	8.03	39.23	4.86
R1 [Ohm∙cm^2^]	9.70 × 10^4^	2.54 × 10^4^	2.68 × 10^4^	1.17 × 10^4^	4.76 × 10^4^	4.24 × 10^4^
C1 [F]	1.10 × 10^−4^	1.38 × 10^−5^	3.02 × 10^−4^	7.90 × 10^−6^	3.22 × 10^−4^	1.27 × 10^−4^
Error [10^−3^]	0.32	0.04	1.63	0.49	0.76	0.57
τ1 = R1 × C1	10.64	0.35	8.09	0.09	15.31	5.36
RINGER						
	**Value**	**SD**	**Value**	**SD**	**Value**	**SD**
Rs [Ohm∙cm^2^]	75.47	8.60	91.71	12.16	71.28	5.67
R1 [Ohm∙cm^2^]	6.94 × 10^4^	5.33 × 10^3^	1.90 × 10^4^	2.18 × 10^3^	1.71 × 10^4^	3.91 × 10^3^
C1 [F]	9.94 × 10^−5^	8.27 × 10^−6^	3.39 × 10^−4^	1.42 × 10^−5^	3.60 × 10^−4^	3.01 × 10^−5^
Error [10^−3^]	0.49	0.07	2.64	0.57	2.51	0.99
τ1 = R1 × C1	6.89	0.04	6.42	0.03	6.16	0.12

**Table 6:** Results of potentiodynamic polarization studies measured in 0.01 M PBS and Ringer solution, where: E_corr_—corrosion potential, I_corr_—corrosion current density, ν_corr_—corrosion rate, R_p_—polarization resistance.

	Ti	Ti98.4–Cu1.6	Ti97Cu3
	0.01 M PBS		
E_corr_ [mV]	−590 ± 32	−634 ± 37	−662 ± 124
I_corr_ [µA/cm^2^]	10.80 ± 2.49	21.94 ± 3.058	26.40 ± 5.53
ν_corr_ [mmpy]	0.041 ± 0.010	0.091 ± 0.013	0.109 ± 0.023
Rp [Ω/cm^2^]	12765 ± 3392	6381 ± 793	4037 ± 1578
χ^2^∙10^−3^	0.26 ± 0.005	0.28 ± 0.018	0.23 ± 0.063
	RINGER		
E_corr_ [mV]	−422 ± 9	−589 ± 10	−675 ± 49
I_corr_ [µA/cm^2^]	7.34 ± 1.16	24.31 ± 1.65	26.49 ± 8.79
ν_corr_ [mmpy]	0.028 ± 0.004	0.101 ± 0.007	0.109 ± 0.036
Rp [Ω/cm^2^]	20249 ± 2561	6431 ± 414	4748 ± 260
χ^2^∙10^−3^	4.14 ± 0.61	5.38 ± 0.58	6.41 ± 3.22

## Data Availability

Data are contained within the article.
